# Strain imaging to assess early effects of successful percutaneous balloon mitral valvotomy on left atrium mechanics

**DOI:** 10.1186/s43044-019-0021-3

**Published:** 2019-11-21

**Authors:** N. Sravan K Reddy, K. Ranjan Shetty, M. Sudhakar Rao, M. Sree Madhurya Reddy

**Affiliations:** 1 Department of Cardiology, Kasturba Medical College Manipal, Manipal Academy of Higher Education (MAHE), Manipal, India; 2 Department of Cardiology, Manipal Hospital, Bengaluru, India; 3 Department of Medicine, Kasturba Medical College Manipal, Manipal Academy of Higher Education (MAHE), Manipal, India

**Keywords:** Balloon mitral valvotomy, Global left atrial strain, Speckle tracking echocardiography

## Abstract

**Background:**

Global left atrial strain (LA) has been used as a novel assessment tool to evaluate left atrial function. However, not much has been investigated to study the effect of percutaneous balloon mitral valvotomy (BMV) in patients with rheumatic severe mitral stenosis on global LA strain. We studied the relationship between global left atrial (LA) strain and severe mitral stenosis and the effect of BMV on LA strain.

**Results:**

A total of 29 patients satisfying the criteria for severe mitral stenosis underwent balloon mitral valvotomy (67% females; mean age, 39.53 ± 11.78 years). Global left atrial strain was assessed by speckle tracking echocardiography before and after valvuloplasty. Global LA strain was impaired in patients with severe mitral stenosis and improved 24–48 h following BMV (13.4 ± .75% vs 17.37 ± 6.95%, *p* < 0.001). There was a significant decrease in mitral mean gradient (MMG) (16.94 ± 6.62 mmHg vs 8.19 ± 4.01 mmHg, *p* < 0.001) and systolic pulmonary artery pressure (sPAP) (47.84 ± 9.07 mmHg vs 36.88 ± 7.69 mmHg, *p* < 0.001) after BMV. Mitral valve area (MVA) (1.045 ± 0.17 cm^2^ vs 1.94 ± 0.22 cm^2^, *p* < 0.001) significantly increased after BMV. Results were compared with 30 age- and sex-matched healthy controls.

**Conclusion:**

Global LA strain can be taken as an indicator of left atrial function, and its improvement following valvotomy may be taken as a good indicator of successful BMV.

## Background

Rheumatic heart disease is the most common acquired cause for mitral stenosis. Rheumatic heart disease causes chordal shortening, commissural fusion, and decrease in mitral valve area. Balloon mitral valvotomy (BMV) is a treatment of choice for rheumatic mitral stenosis.

Mitral valve is the most commonly involved valve in rheumatic heart disease and usually presents with exertional dyspnea, pulmonary hypertension, and right heart failure. Exertional dyspnea occurs secondary to increased left atrial pressure leading to increase in pulmonary capillary wedge pressure. Severe mitral stenosis leads to left atrial enlargement leading to LA longitudinal lengthening which is recorded as positive strain. Quantification of global LA strain by two-dimensional speckle tracking echocardiography is a diagnostic tool for assessment of left atrial function. This study was done to assess LA function by global LA strain and its reversibility following balloon mitral valvotomy.

## Methods

We performed a prospective case control study from December 2014 to November 2016. Twenty-nine patients satisfying the inclusion criteria were studied and underwent detailed echocardiographic assessment pre- and post-balloon mitral valvotomy. Sample size was calculated with alpha of 1%, clinical significant difference (d) of 2, and power of 90% using the following equation:

$$ ``n-2\left(z1-\alpha /2+z1-\upbeta \right)2\ \delta 2/\mathrm{d}2" $$

A sample size of 29 was reached based on the above formula.

Patients with severe rheumatic mitral stenosis in normal sinus rhythm with valve suitable for BMV and who underwent successful BMV were included in the study. Patients in atrial fibrillation (during hospitalization for BMV or history of AF or paroxysmal AF), more than mild mitral regurgitation (MR), aortic regurgitation (AR), diabetes mellitus, hypertension, and renal failure were excluded from the study. Control group included 30 age- and sex-matched healthy volunteers.

### Balloon mitral valvotomy

Indications for BMV in mitral stenosis included symptomatic severe mitral stenosis (New York Heart Association class II–IV and mitral valve area calculated by planimetry ≤ 1.5 cm^2^), less than grade 2+ mitral regurgitation, and favorable morphology of mitral valve. All patients underwent balloon mitral valvuloplasty using Inoue balloon method. Successful BMV procedure was defined as achieving either a final MVA > 1.5 cm^2^ or increase in MVA by 40% and mitral regurgitation grade ≤ 3+ [1]. None of the patients who underwent BMV had any peri-procedural or post-procedural complications.

### Echocardiographic examination

The measurements were performed echocardiographically using the Philips Epiq 7c system. All the parameters were taken by a single person to avoid observer bias. The area of mitral valve was calculated using 2D planimetric method. Continuous-wave Doppler was used to measure the gradients across the mitral valve and pulmonary artery systolic pressure.

Post-BMV mitral valve area, pulmonary artery pressure, mean mitral gradient, and global LA strain were measured 24–48 h after procedure. Delta (Δ) was used to define the absolute changes in the valve area and gradients across the mitral valve pre- and post-BMV.

$$ {\displaystyle \begin{array}{l}\varDelta \mathrm{Mitral}\ \mathrm{valve}\ \mathrm{area}=\mathrm{post}-\mathrm{BMV}\ \mathrm{MVA}\ \mathrm{value}-\mathrm{pre}-\mathrm{BMV}\ \mathrm{MVA}\ \mathrm{value}\\ {}\varDelta \mathrm{Mean}\ \mathrm{mitral}\ \mathrm{gradient}=\mathrm{post}-\mathrm{BMV}\ \mathrm{MMG}\ \mathrm{value}-\mathrm{pre}-\mathrm{BMV}\ \mathrm{MMG}\ \mathrm{value}\end{array}} $$

#### Speckle tracking

For speckle tracking, apical four-, three-, and two-chamber views were obtained using standard 2D gray scale echocardiography with breath hold and stable electrocardiographic recording. The average of three cardiac cycles was recorded. The frame rate was set at 60–80 frames/s. These settings are recommended to combine temporal resolution with adequate spatial definition and to enhance the probability of the frame-frame tracking technique [2]. During offline image analysis of 2D cine loops with deformation study, 2D left atrial wall in apical four-, two-, and three-chamber views were tracked in semi-automated method. Offline analysis of the recorded images was analyzed by a single experienced echocardiographer who was not involved in image acquisition and had no knowledge of other echocardiographic variables using Philips automated cardiac motion quantification (ACMQ) software.

**Fig. 1:**
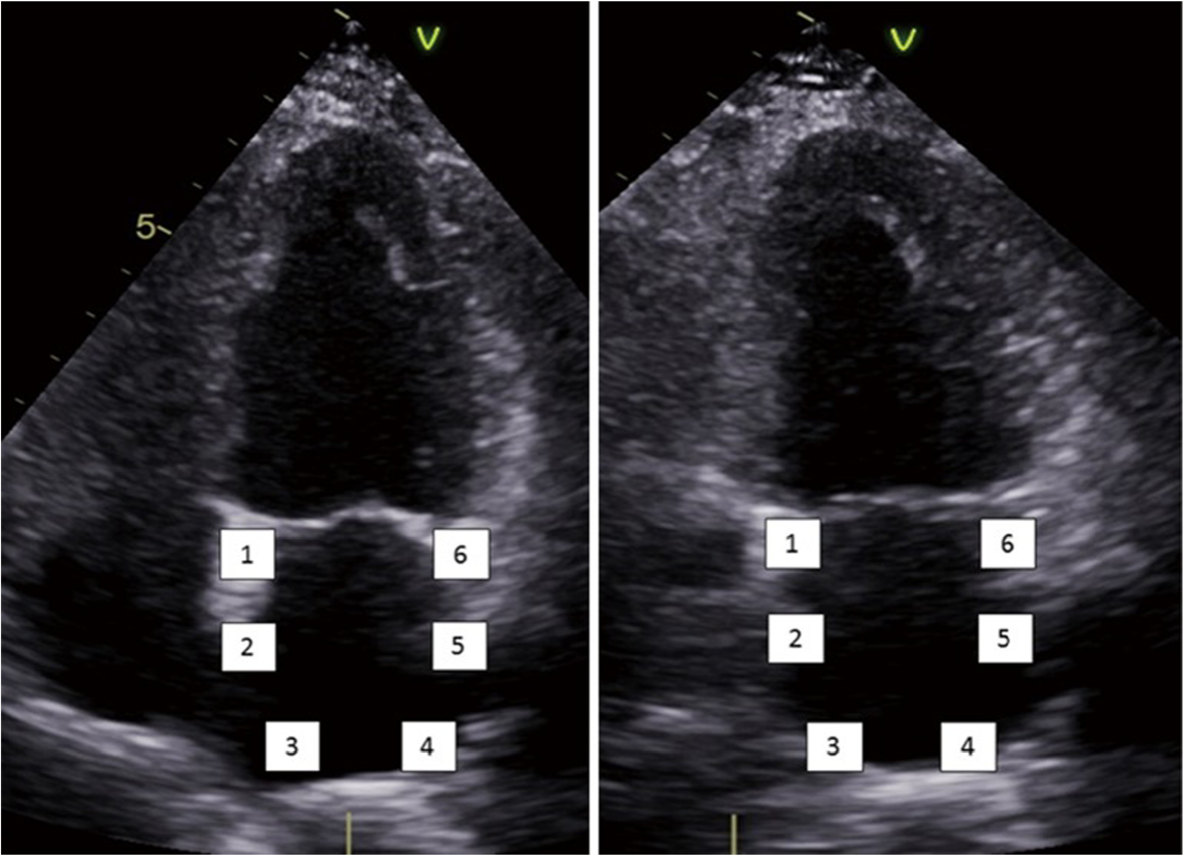
Apical four-chamber and two-chamber views of the six left atrial segments

**Fig. 2:**
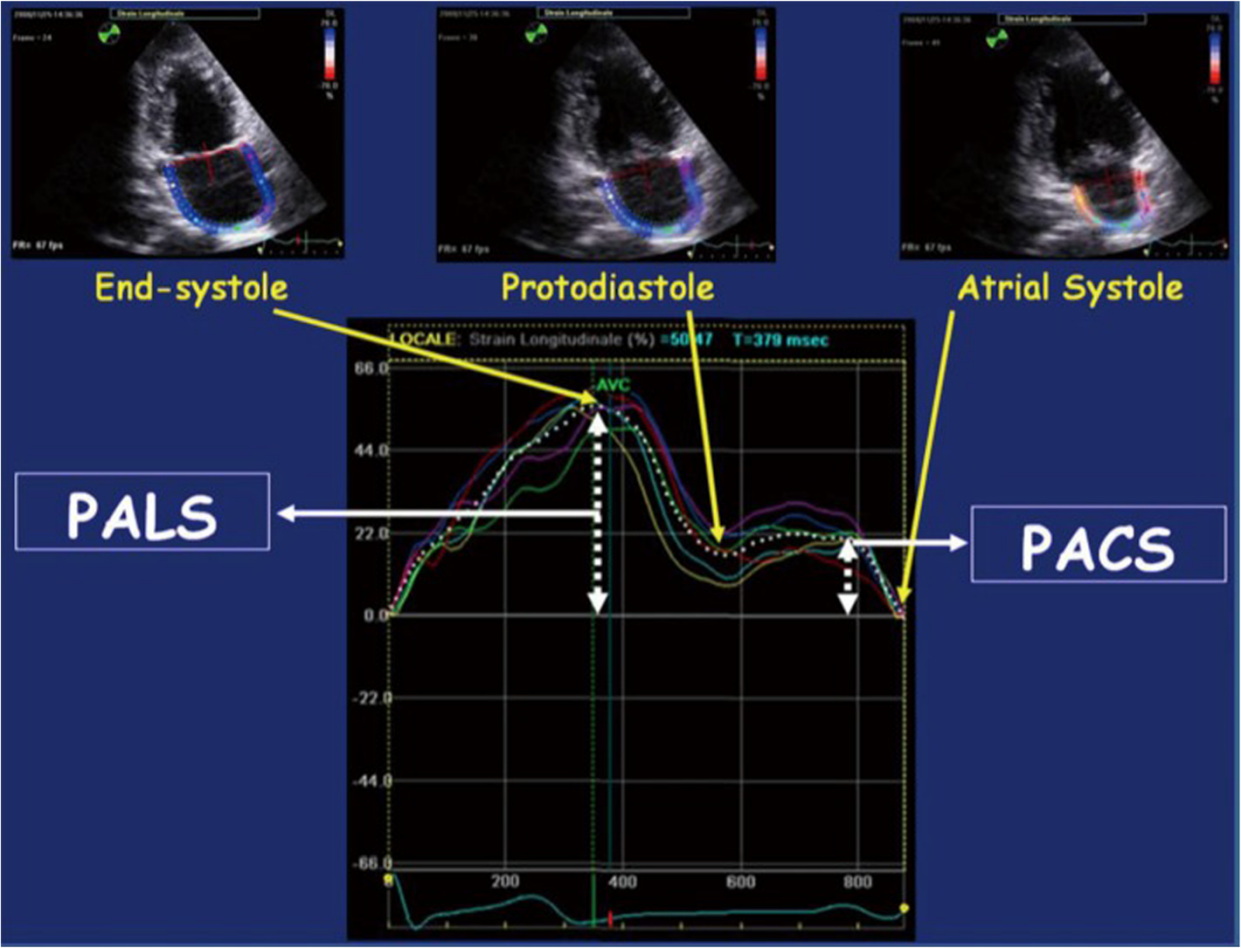
Peak atrial longitudinal strain (PALS) and peak atrial contraction strain (PACS). Modified from Cameli et al. [3]

First, the endocardial left atrial surface was traced manually using the point and click method in apical four-, three-, and two-chamber views, following which epicardial surface was traced automatically by the software system creating a region of interest involving the entire myocardial thickness. Lastly, the system generates two strain curves for each atrial segment. In total, 12 segments were analyzed after acquiring decent image quality (Fig. 1). In sinus rhythm, atrial strain shows two distinct wave forms, peak atrial longitudinal strain (PALS) and peak atrial contraction wave. In the present study, we measured global PALS, i.e., average of PALS in all segments of left atrium (Fig. 2). According to the current American Society of Echocardiography/European Association of Echocardiography Consensus, the global positive PALS was measured at the end of the reservoir phase using a 12-segment model and QRS onset as the reference point before and after the procedure.

### Statistical analysis

SPSS.v.16.0 software was used for analyzing statistics. Continuous variables were expressed as mean +/− SD. Categorical data were analyzed using chi-square test. Differences between groups (case vs control) were evaluated with independent *t* test. Differences between the cases (pre-BMV vs post-BMV) were compared using paired *t* test. *p* value, i.e., level of statistical significance, was set at < 0. 05.

## Results

**Fig. 3:**
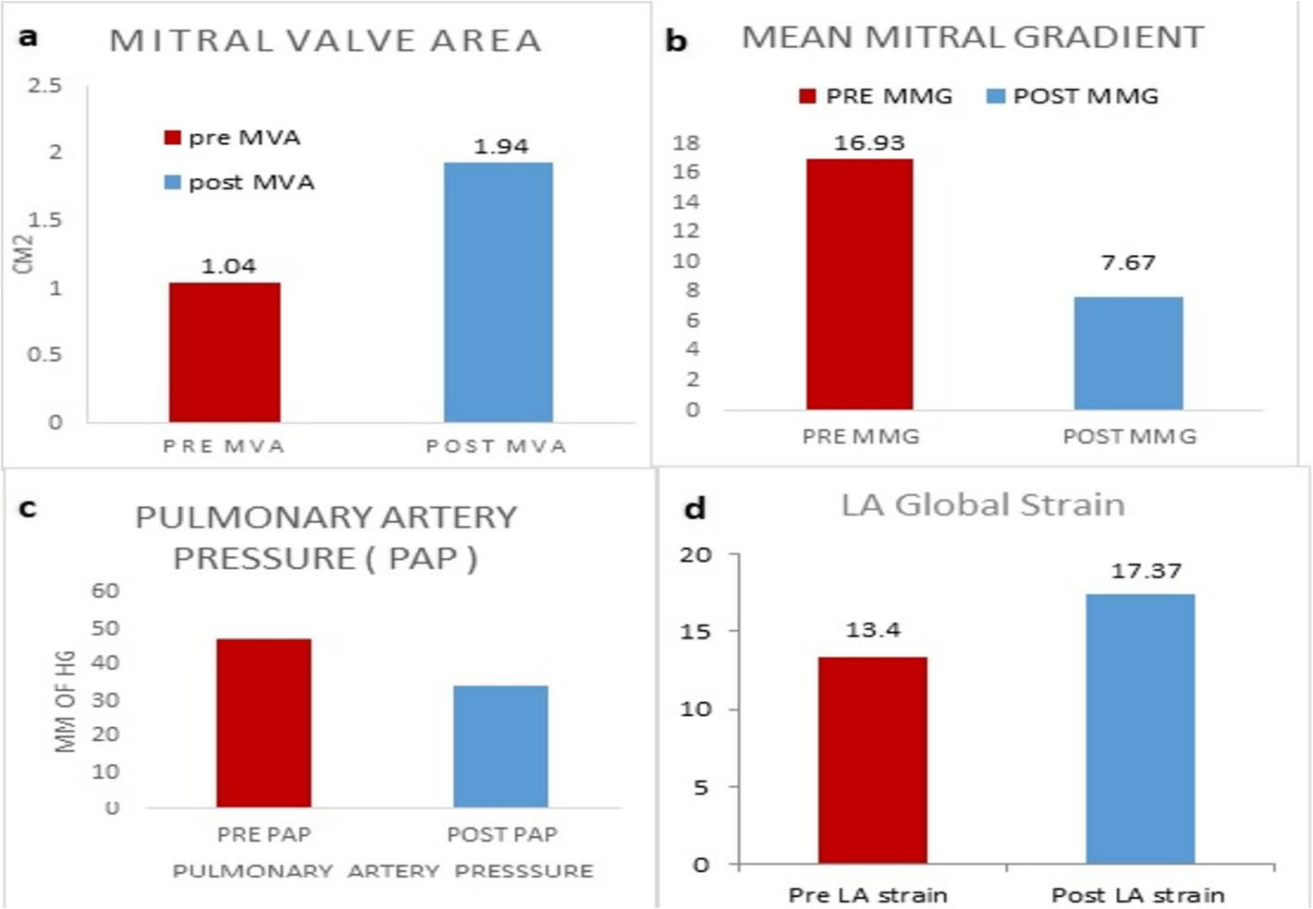
Comparison of various echocardiographic parameters of pre- and post-BMV in mitral stenosis. **a** Mitral valve area. **b** Mean mitral gradient. **c** Pulmonary artery pressure. **d** LA global strain

From December 2014 to November 2016, 29 patients satisfying the inclusion criteria were included in the study and underwent detailed echocardiographic assessment pre- and post-balloon mitral valvotomy. The patients’ baseline clinical and echocardiographic profiles are shown in Table 1 and Fig. 3.

**Table 1:** Clinical and trans-thoracic echocardiographic findings before and after balloon mitral valvotomy

	Mitral stenosis (*n* = 29)	Control group (*n* = 30)	Post-BMV (*n* = 29)	P1 value	P2 value
Age	39.53 ± 11.78	44.27 ± 6.83	–	0.06	–
SBP (mmHg)	113.38 ± 14.66	123.20 ± 9.90		0.003	
DBP (mmHg)	75.00 ± 8.27	78.67 ± 5.49		0.043	
LA (IS in mm)	64.37 ± 9.94	42.87 ± 5.44		0.001	
LA area (in cm^2)^	28.36 ± 8.28	11.94 ± 1.96		0.001	
LA strain (%)	13.40 ± .75	32.25 ± 4.02	17.37 ± 6.95	0.001	0.001
EF(%)	62.40 ± 5.10	61.50 ± 4.50	62.40 ± 5.10	0.48	–

Plus-minus values are means ± SD. Difference between the groups were analyzed by independent *t* test

*BMV* balloon mitral valvotomy, *SBP* systolic blood pressure, *DBP* diastolic blood pressure, *LA (IS)* left atrial inferosuperior diameter, *mm* millimeters, *cm* centimeters, *EF* ejection fraction, *P1* mitral stenosis vs control, *P2* post-BMV vs control, *EF* ejection fraction

Our study included 29 adult patients with mitral stenosis as well as 30 healthy controls (mean age, 39.53 ± 11.78 years vs 44.27 ± 6.83 years, *p* = 0.06; females, 69% vs 66%, *p* = NS) (Additional file 1). Transthoracic echocardiography findings of pre-BMV and 24–48 h post-BMV are shown in Table 2. There was no significant difference in age and ejection fraction between mitral stenosis and control groups; however, systolic blood pressure and diastolic blood pressure were significantly higher in the MS group compared with the controls. Mean left atrial diameter and left atrial area was significantly higher in the MS cases compared with the controls. Baseline left atrial strain was significantly lower in patients with mitral stenosis compared with the controls.

**Table 2:** Transthoracic echocardiographic and 2D STE findings before and after balloon mitral valvotomy

	Pre-BMV	Post-BMV	*p* value
MVA (cm^2^)	1.045 ± 0.17	1.94 ± 0.22	< 0.001
MMG (mmHg)	16.94 ± 6.62	8.19 ± 4.01	< 0.001
PAP (mmHg)	47.84 ± 9.07	36.88 ± 7.69	< 0.001
LA strain (%)	13.40 ± .75	17.37 ± 6.95	< 0.001

Plus-minus values are means ± SD. Difference between pre- and post-BMV values were analyzed using paired *t* test

*BMV* balloon mitral valvotomy, *MVA* mitral valve area calculated using planimetry, *MMG* mean mitral gradient, *PAP* pulmonary artery pressure, *LA strain* left atrial strain

The mitral valve area increased and the mean mitral gradients decreased significantly post-BMV (1.045 ± 0.17 mm^2^ vs1.94 ± 0.22 mm^2^, 16.94 ± 6.62 mmHg vs 8.19 ± 4.01 mmHg).

Left atrial global strain was significantly impaired in mitral stenosis. However, following BMV, the left atrial global strain significantly improved compared with pre-BMV (13.4 ± .75% vs 17.37 ± 6.95%, *p* < 0.001) (Table 2).

## Discussion

Rheumatic carditis causes left atrial dilatation, myocyte necrosis, interstitial fibrosis, and disorganization of atrial muscle bundles. This structural remodeling impairs both contraction and relaxation functions of atrial myocytes. Also, narrowing of mitral valve secondary to stenosis leads to increased afterload of the left atrium. These structural and hemodynamic changes cause progressive impairment of left atrium mechanical function [4, 5].

The left atrium mechanical function assessed by echocardiography can either be load dependent or load independent. Conventionally, atrial function is usually assessed by atrial volumes which is load and operator dependent and does not accurately evaluate atrial reservoir function. Two-dimensional transthoracic speckle tracking echocardiography (2D STE) allows the assessment of global left atrium mechanic [6–8]. Cardiac deformation assessment is the most reliable and reproducible method of assessing ventricular and atrial function with the advantage that it is a load-independent parameter which depicts myocardial function. Till now, no validated algorithms have been developed exclusively for the evaluation of left atrial function. Many studies have utilized strain software that was developed for the left ventricle, with adjustments to the width of the “region of interest” to evaluate left atrial strain. Filling and stretching of the left atrium occurs in the reservoir phase which causes positive atrial strain reaching its peak in systole at the end of left atrial filling, prior to the opening of mitral valve. In the next phase, passive left atrial emptying starts with the opening of the mitral valve resulting in decreased atrial strain causing negative deflection of the strain curve up to a plateau period which is analogous to diastasis phase. A second deflection in the strain curve is then observed corresponding to atrial systole. Peak atrial longitudinal strain (PALS) is measured at the end of the reservoir phase. Peak atrial contraction strain (PACS) is measured following the P wave and represents active atrial contraction [3].

Though many studies have been done studying the impact of balloon mitral valvotomy on atrial volumes, atrial pumping function, and atrial reservoir functions [9–11], not many studies have studied the effect of valvotomy of global left atrial strain. Rohani et al. studied the acute effect of balloon mitral valvotomy and mitral valve replacement in patients with mitral stenosis and concluded that the peak atrial longitudinal strain (PALS) was impaired in patients with severe symptomatic mitral stenosis and improved acutely after treatment [12]. Hemodynamic changes following BMV include decrease in left atrium afterload which is reflected by an increase in mitral valve area, decrease in diastolic transmitral gradients, and decrease in systolic pulmonary artery pressure. In this study, there was a significant reduction in the left atrial strain post-balloon mitral valvotomy; however, it did not reach normal value. So, it needs to be further studied if the left atrial strain further reduces during mid- and long-term follow-up and also if this correlates with the reduction in atrial fibrillation and thrombus formation.

The present study has the following limitations. It was a non-randomized study. Also, the prognostic effect of speckle tracking echocardiography following balloon mitral valvotomy was not evaluated as patients were not followed up. We did not study the effect of balloon mitral valvotomy on either right or left ventricular strain, nor study the left atrium active emptying fraction for patients with mitral stenosis before and after valvotomy.

## Conclusion

Global LA strain can be taken as an indicator of left atrial function, and its improvement following valvotomy may be taken as a good indicator of successful BMV. However, whether this improvement in left atrial strain leads to a reduction in rate of atrial fibrillation during mid- and long-term follow-up needs further studies.

## Supplementary information

**Additional file 1:** Raw data from patients with mitral stenosis before and balloon mitral valvotomy and in controls.

## Data Availability

Submitted as supplementary file.

## Abbreviations

BMV: Balloon mitral valvotomy
MMG: Mean mitral gradient
MR: Mitral regurgitation
MVA: Mitral valve area
PAP: Pulmonary artery pressure
STE: Speckle tracking echocardiography
